# Attitudes and beliefs of Australian chiropractors’ about managing back pain: a cross-sectional study

**DOI:** 10.1186/s12998-015-0062-y

**Published:** 2015-05-11

**Authors:** Stanley I Innes, Peter D Werth, Peter J Tuchin, Petra L Graham

**Affiliations:** Discipline of Chiropractic, Health Professions, Murdoch University, Murdoch, WA 6150 Australia; Private Practice, Australian Injury Management Consulting, 117 Hall Road, Carrum Downs, VIC 3201 Australia; Department of Chiropractic, Faculty of Science, Macquarie University, Sydney, NSW 2109 Australia; Department of Statistics, Faculty of Science, Macquarie University, Sydney, NSW 2109 Australia

**Keywords:** Low back pain, Chiropractic, Attitudes and beliefs, Biopsychosocial

## Abstract

**Background:**

Chiropractors are frequent providers of care for patients with lower back pain. Biopsychosocial approaches to managing patients are regarded as best practice and are gaining wider acceptance. Recent evidence suggests that practitioners’ attitudes and beliefs may also have an important effect on patients’ recovery from back pain. Past studies have pooled manual therapists from differing professions. Dissonant findings have been hypothesised as being a result of the chiropractic subpopulation within multi-practitioner participant pools who are hypothesised to focus on biomedical aspects of treatment and minimize biopsychosocial dimensions.

The aim of this study is to determine whether a study population of only chiropractors would demonstrate similar attitudes and beliefs to other manual therapists’ biopsychosocial or biomedical approach to the management of their patients.

**Methods:**

A survey of chiropractors in Victoria Australia in September 2010 was undertaken utilising the Pain Attitude and Belief Scale (PABS.PT), a tool which has been developed to determine the orientation (biopsychosocial or biomedical approach) of practitioners to the management of people with low back pain. The survey also obtained demographic data from respondents to determine whether variables such as education, gender or practice related factors influenced their orientation.

**Results:**

The overall response rate was 29% (n = 218). The majority of the sample was male (68%), with a mean age of 44 years. The 6 point Likert scale scores were 34.5 (6.3) for the biomedical factor scale and 31.4 (4.1) for the biopsychosocial scale. Internal consistency of the psychosocial subscale was poor. None of the demographic variables were found to influence the biomedical or psychosocial scales.

**Conclusions:**

Chiropractors in the state of Victoria were found to have similar biomedical and psychosocial orientations in their attitudes and beliefs when compared to other manual therapists’ levels of previous studies from differing cultural and educational backgrounds. This study was unable to replicate any of the relationships from past studies with any of the demographic variables. The psychosocial scale internal consistency may be a significant factor in this non-finding. Future research should address the identification of more robust items of the biopsychosocial attitudes of Victorian chiropractors toward treating lower back pain.

## Background

Low back pain is now the leading cause of disability globally and was estimated to be responsible for 83 million years lived with disability in 2010 [1]. Manual therapists, such as chiropractors, physiotherapists and osteopaths, are a significant consumer choice for the treatment and management of this condition. During 2005, in Victoria Australia, over 3 million people sought care at least once from a chiropractor [2]. While a recent study has documented the type of conditions chiropractors encounter, there has been little research exploring therapists’ attitudes and beliefs which may improve patient outcomes and thus reduce levels of associated disability [3].

It is now well established that the problem of persistent low back pain and associated levels of disability are not fully explained by biological factors alone and that best practice care should include more than the musculoskeletal system. There is good evidence that psychological constructs such as pre-existing depression, anxiety, fear-avoidance beliefs, poor coping strategies and poor self-efficacy are significant predictors of greater functional disability and work loss [4]. These constructs appear to play an important role in the transition from the acute setting to persistent pain and disability. Evidence also suggests that social and organisational factors may play a role in these negative outcomes, though the exact mechanism remains unclear [5]. Consequently this investigation sought to expand the understanding of chiropractic interventions by studying these non-mechanical dimensions.

The most dominant current model in understanding these is the biopsychosocial model (BPSM) of health. It describes the influence of the biological, psychological and sociological factors in the manifestation of pain and illness [6]. It may best be considered as a heuristic to better understand these dimensions of care [7]. There are now validated outcome measures stemming from BPSM research that enhances the primary contact practitioner’s ability to detect in the early phases this transition into disability [8]. The practitioner is thus able to avoid inappropriate and ineffective passive modalities of care and implement more appropriate interventions [9].

Despite the BPSM recently celebrating its 25^th^ birthday not all manual therapists are cognisant of the impact of the psychological and sociological domains. In response to this various government and insurance agencies have attempted to implement readily applicable assessment frameworks and education programs to improve patient outcomes [10]. Despite these efforts there has been no appreciable diminution of the rates of disability or its financial impost. Psychological and sociological interventions to date have only produced modest changes at best [11]. Subsequently researchers are now beginning to explore other facets of the BPSM.

One line of exploration that researchers have turned their focus toward is that of the attitudes and beliefs of the practitioner [12]. A systematic review of the relationship between practitioners’ beliefs and behaviours concluded that there is moderate evidence that practitioners’ fear-avoidance beliefs are associated with reported sick leave prescription thus directly influencing patient’s behaviour [13]. For example, a practitioner may be overly anxious about the patient exacerbating a lower back injury and may unnecessarily recommend reduced activity, bed rest and time away from work, thus reinforcing pain behaviours. Several studies have now been conducted to explore the possibility of measuring these attitudes and beliefs in various manual therapy groups [14-17].

One instrument used to explore these attitudes is the Pain Attitudes and Beliefs Scale for Physiotherapists (PABS.PT) [16,17]. It was developed in an attempt to identify the extent to which practitioner attitudes and beliefs in two domains; biomedical and/or psychosocial which impact on patients response to lower back pain. While the developers of the PABS.PT state that these domains do not sit on a continuum, several studies have shown a low negative correlation [18-20]. A practitioner who scores highly on the biomedical orientation is thought to have a strong belief that there is a relationship between pain and tissue damage. Those who attain higher scores on the psychosocial dimension are thought to be more likely to believe that lower back pain outcomes are a result of tissue damage and can be influenced by social and psychological factors. Thus it may be possible to identify those practitioners by their high scores on biomedical orientation and low psychosocial orientation who are less likely to follow evidence-based guidelines best quality care and can be assisted with further education interventions to improve the quality of care delivery [18].

The initial version PABS.PT was comprised of 31 items which were extracted from existing psychosocial related questionnaires by an expert physiotherapist panel [16]. Factor analysis revealed two discrete factors: “Biomedical Orientation or factor 1” and “Behavioural Orientation or factor 2” which explained 25% and 8% of the variance respectively. “Biomedical Orientation” demonstrated adequate internal consistency (Cronbach’s alpha 0.83) but the “Behavioural Orientation” obtained a Cronbach’s alpha of 0.54. Subsequently the authors suggested future research into the items of factor 2 in order to improve its psychometric properties. They recommended the recruitment of practitioners from a variety of professions (orthopaedic surgeons and chiropractors) in order to achieve extreme scores. It was noted that women tended to score higher on both scales than men and men of greater than 42 years of age scored significantly higher on the “Behavioural factor”.

Houben et al. [17] continued the psychometric development of the PABS.PT and followed these recommendations by recruiting 295 Dutch physiotherapists, manual therapists and chiropractors. They also selected 5 additional items based on “face value” and added these to the original PABS.PT. These additional items were aimed at enhancing the second factor. Statistical analysis produced a 19 item inventory with 2 factors closely resembling those of Ostelo et al. [16]. Factor 1 (Biomedical Orientation) was best described by 10 items and explained 23.4% (Cronbach’s alpha 0.80) of the variance. Factor 2 (BPS orientation) was best described by 9 items and these explained 10% (Cronbach’s alpha 0.68) of the variance. No differences were observed on the factors with regard to gender, age or years of work experience. Chiropractors scored significantly lower on factor 2 compared to the other treatment disciplines. Chiropractors also obtained the highest score on the Biomedical factor but this did not reach a level of significance.

Subsequent studies have verified the two factor structure of the PABS.PT [18-21]. These studies have also explored other dimensions and related factors. A significant linear trend of increasing disparity with treatment guidelines as biomedical scores increased and behavioural scores decreased was found in a United Kingdom study who recruited 1012 general practitioners (GPs) and physical therapists [19]. Irish GPs low biomedical scores were more likely to be found in those who were more recently qualified and more likely to follow best practice guidelines, however the authors removed 9 items from the Houben et al. version of the PABS.PT to improve the internal consistency of the questionnaire [18]. Brazilian male physiotherapists and those with less experience were significantly more likely to follow a biomedical approach to the treatment of patients with chronic lower back pain [21]. It should be noted that this Brazilian study summed the short form of the PABS.PT on a 0 = “totally disagree” and 5 = “totally agree” Likert scale as opposed to a “1 through 6” Likert scale standard in prior studies. This Brazilian study, when discussing differences in mean scores on the PABS.PT between previous studies suggested that the high scores could possibly be a result of a group of professionals such as chiropractors who would be expected to have a stronger biomedical orientation.

This suggestion of the chiropractic population possessing different qualities to other health professionals is not without precedent. For example the STarT back screening tool had been developed to help primary care practitioners make care decisions about the likely need LBP patients have for secondary prevention based on modifiable risk factors for poor outcomes [22,23]. It had been validated with physiotherapists and medical practitioners but was unable to be replicated with chiropractors in the UK [24]. The authors suggested several possibilities for this outcome. First that this was due to patient population differences as these were a self-selecting population who sought chiropractic care privately and as such had a different psychological profile. Other reasons included higher expectations of a positive outcome. They also mused that differences in treatment approaches used by chiropractors could be a factor. Finally they suggested that chiropractors may have consciously or unconsciously addressed the patients’ psychological needs. In contrast, a Norwegian study has suggested that psychological factors are not relevant in the prediction of treatment outcomes in chiropractic patients as psychosocial issues constitute a negligible portion of their daily encounters [25].

Other possible explanations for this professional dissonance include the presence of a significant portion who hold unorthodox views [26], a limited research capacity, a lower value placed on scientific knowledge, a short history with decision support systems such as guidelines and a lack of developed coordinated efforts to address low coherence of beliefs and evidence-based practices [27].

Therefore, the primary aim of this study was to further explore chiropractors’ attitudes and beliefs in an effort to understand these reported differences. In particular it sought to measure and compare their attitudes and beliefs in regard to biomedical and psychosocial aspects of patient care in a sample of only chiropractors in Victoria, Australia. A secondary aim was to determine if sociodemographic characteristics may be also be associated with their beliefs and attitudes.

## Methods

The study consisted of a retrospective survey of chiropractors practising in the state of Victoria Australia in October 2010. Ethical approval was obtained from Human Research Ethics Committee of Macquarie University, New South Wales, Australia (HE25SEP2009-ROO143). All questionnaire responses were completed anonymously, thus written consent was not obtained from individual respondents with completion of the questionnaire indicating consent.

### Instruments

The 19 item shortened version PABS.PT was used to evaluate the role of chiropractors’ attitudes and beliefs on the development and maintenance of persistent low back pain [17]. Previous studies have produced and validated the presence of 2 discrete scales. The Biomedical scale consists of 10 items which are scored on a 6 point Likert scale (1=”totally disagree” to 6 “totally agree”) and are summed to produce a score between 0 and 60 points. A high score indicates a belief in the relationship between low back pain and tissue damage. The Psychosocial scale consists of 9 items and is likewise summed to produce a score between 0 and 54. A high score indicates a belief in the influence of psychological, social and biological factors.

A recently published systematic review of the psychometric properties of the PABS.PT found positive results for its construct validity, reliability and responsiveness [20].

### Sample

A mailing list of all registered chiropractors in the state of Victoria was obtained from the national chiropractic registration board. Duplicate names, incomplete details and practitioners who were not practising in Victoria and returned mail from practitioners who were no longer practising at the designated address were excluded from the sample. This resulted in a list of 750 eligible practitioners.

### Survey dissemination

All 750 practitioners were mailed an invitation letter which consisted of the PABS.PT and 10 demographic questions. The survey was accompanied by a reply paid envelope to return to the authors. Alternatively, practitioners were given the opportunity to complete the survey online via an online survey tool (Survey Monkey™). A reminder was forwarded to practitioners via electronic mail with the assistance of the professional associations.

### Statistical methods

Data were analysed with SPSS V21. Descriptive statistics were calculated for the demographic items and Cronbach’s alpha was used to examine internal consistency of the questionnaire items.

Median scores on the biomedical and psychosocial factors were calculated and the sample was dichotomised using these values. Logistic regression was then used to determine whether any demographic variables (age in years, gender, number of patients per week, years working chiropractor, whether or not post graduate studies had been undertaken, urban or rural practice location, type of chiropractic national body membership) could explain the dichotomisation (i.e. whether or not the chiropractors have higher odds of the particular domain tendency).

In order to compare this sample to the national population, figures were obtained from the Australian Workforce Data Analysis and Planning, Department of Health [28]. These data were only available in 10 year age ranges.

The mean and standard deviation scores for the biomedical and psychosocial scales were calculated on a “1 to 6” Likert scale and a “0 to 5” Likert scale to allow for comparison to the Magalhães et al., [14,21] study.

A multiple regression analysis was used with the biomedical and psychosocial scale scores as the dependent variables and the demographic factors were entered as independent variables to test for the presence of independent predictors.

## Results

The overall response rate to the survey was 29% (n = 218). Demographic data are summarised in Table 1. The average age of the chiropractors in this study was 44 years of age, males constituted the majority of the sample (68%) and had been in practice for an average of 17.5 years. They worked 30 hours per week, consulting 90 patients per week and approximately one third held post graduate degrees.

**Table 1:** **Demographic data of Victorian chiropractic participants and national demographic data (where available)**

	**(n = 218)**	**National workforce (4854)**
Gender (% female)	69 (31.7)	479 (35.7)
Age in Years: 16-34	18.9%	35.6%
35-44	40.0%	30.4%
45-54	24.0%	18.4%
55-64	14.0%	10.7%
65-74	2.2%	4.3%
75-89	-	0.8%
Practice type (%)		
Group	47.9%	54%
Solo	52.1%	46%
Location (%)		
Metro	67.1%	75%
Rural	32.4%	24%
Remote	0.5%	1%
Postgraduate qualifications	32.7%	n/a
Professional Association (%)		
CAA	122 (56.2)	n/a
COCA	59 (27.2)	n/a
CAA and COCA	15 (6.9)	n/a
Other	8 (3.7)	n/a
None	13 (6.0)	n/a
Average number of years in practice (SD)	17.5 (9.2)	13.1
Average hours per week in practice (SD)	30.3 (11.6)	n/a
Median number of patients per week (range, SD)	90 (0-380)	n/a
**Years worked**		
0-5	17.3	25.6
6-10	23.7	18.3
11-15	22.5	13.6
16-20	15.6	9.2
21-25	13.3	7.6
26 or more	7.5	14.6
**Hours Per Week**		
1-10	4.2	9.6
11-15	10.0	7.6
16-20	2.6	9.0
21-25	14.7	10.0
26-30	20.5	16.9
31-35	17.9	14.4
36-40	15.8	15.3
41-45	5.8	3.9
46-50	5.8	3.2
51-55	1.6	0.9

The average 6 point Likert scale scores were 34.5 (6.3) for the biomedical factor and 31.4 (4.1) for the biopsychosocial scores. The 5 point Likert scale produced biomedical factor scores of 24.7 (6.3) and a biopsychosocial score of 22.4 (4.1) (Table 2).

**Table 2:** **Means and standard deviations of PABS.PT past studies scores**

**Study**	**PABS.PT**_**biomedical**_		**PABS.PT**_**biopsychosocial**_	
	**Score**	**SD**	**Score**	**SD**
Innes *et al (1-6 Likert scale)*	34.5	6.3	31.4	4.1
Houben *et al, 2005* [17]	29.5	7.9	35.6	5.6
Bishop *et al, 2008* [19]	31.1	7.2	32.5	4.8
Fullen *et al, 2011 (10 items version)*	38.8	7.7	16.3	3.1
**Magalhães** ***et al, 2011*** [14,21]	**27.6**	**7.2**	**24.3**	**6.3**
**Innes** ***et al (1-5 Likert scale)***	**24.7**	**6.3**	**22.4**	**4.1**

The biomedical subscale Cronbach’s alpha reached an acceptable level of 0.74 and the psychosocial subscale was 0.42.

The biomedical and psychosocial scales were inversely related as shown in several previous studies (*r* = -0.27).

Logistic regression analysis indicated no evidence that any of the demographic variables helped to predict domain tendency (*p* > 0.140, results not presented). Multiple linear regression analysis demonstrated that the chiropractic demographic variables accounted for 12% of the variance of the biomedical factor (F (8, 131) = 1.210, *p* = 0.298, R^2^ = 0.12) and 20% of the psychosocial factor (F(8,131) = 1.348, *p* = 0.226, R^2^ = 0.20) (Tables 3 and 4). Only the number of patients for the psychosocial scale approached significance at the 5% level (beta coefficient = 0.175; 95% CI: -.0003 to 2.704; p = 0.05). Sample size adequacy to perform the regression analysis was calculated with GPower 3.1. With a sample size of 218 participants and 8 independent variables, 99.6% power was obtained to detect a low correlation.

**Table 3:** **Multiple regression analysis of PABS.PT psychosocial (dependent variable) with sociodemographic variables**

**Coefficients**								
**Model**		**Unstandardized coefficients**		**Standardized coefficients**	**t**	**Sig.**	**95.0% confidence interval for B**	
		**B**	**Std. error**	**Beta**			**Lower bound**	**Upper bound**
1 (Constant)		34.765	2.335		14.891	.000	30.146	39.383
Patients		1.351	.684	.175	1.974	.050	−003	2.704
Years_Prac		.051	.077	.120	.656	.513	−102	.204
Age		−095	.072	−249	−1.320	.189	−236	.047
PostGrad		−092	.711	−011	−129	.897	−1.499	1.315
Location		−801	.703	−101	−1.139	.257	−2.193	.591
PracType		−749	.692	−097	−1.082	.281	−2.118	.620
ChiroAssoc		−244	.772	−028	−317	.752	−1.772	1.283
Gender		.104	.775	.013	.134	.894	−1.430	1.637

**Table 4:** **Multiple regression analysis of PABS.PT biomedical (dependent variable) with sociodemographic variables**

**Coefficients**								
**Model**		**Unstandardized coefficients**		**Standardized coefficients**	**t**	**Sig.**	**95.0% confidence interval for B**	
		**B**	**Std. error**	**Beta**			**Lower bound**	**Upper bound**
1(Constant)		35.346	3.831		9.226	.000	27.768	42.924
Patients		−1.917	1.115	−153	−1.719	.088	−4.122	.289
Years_Prac		−031	.127	−045	−244	.808	−283	.221
Age		.019	.118	.031	.162	.871	−214	.252
PostGrad		−1.475	1.154	−108	−1.278	.203	−3.757	.807
Location		−1.442	1.144	−112	−1.261	.210	−3.705	.820
PracType		1.482	1.131	.118	1.311	.192	−755	3.719
ChiroAssoc		1.296	1.263	.092	1.026	.307	−1.202	3.795
Gender		.288	1.254	.022	.229	.819	−2.193	2.769

## Discussion

This study's aim was to determine if chiropractors' attitudes and beliefs regarding the management of patients with back pain were consistent with previous findings where a contemporary biopsychosocial approach or a biomedical approach has been shown to impact on the management of back pain when delivered by first contact practitioners. Anomalous findings in past studies suggested that the chiropractic subpopulation may be a cause of experimental artefact [20,24]. This is the first study to investigate the attitudes and beliefs of chiropractors in isolation.

The chiropractic sample in this study, when compared to the national chiropractic workforce data, had similar proportion of female chiropractors and average hours per week worked. However several differences were noted. The current study had a smaller portion of chiropractors participating in group practice compared to the national figures (48% versus 52%) and were less represented in the younger age groups (16 -34 years) compared to the national figures (18.9% versus 35.6% respectively). Chiropractors in this study tended to have more work experience in the 10 to 20 years range (38% compared to 21%). So this sample may be over represented by “mid-life” chiropractors.

The results of this study showed that the estimated chiropractic population’s scores on the two subscales of the 19 item PABS.PT were within the ranges of previous studies. Scores were calculated on a “0-5” Likert scale for comparison to the Brazilian chronic pain physiotherapists in Magalhães et al. [14,21] and were also of a similar magnitude (biomedical = 27.6, psychosocial = 24.3 compared to this study of 24.7 and 22.4 respectively). Calculating means and SD using the “1 – 6” Likert scale, Victorian chiropractors were most similar to the United Kingdom GPs and physiotherapists [19] and least similar to Irish GPs [18]. The findings of this study do not support the hypothesis that chiropractors have an extreme tendency towards biomedical or biopsychosocial orientations as proposed by Magalhães et al. [14,21].

Magalhães et al. [14,21] raise several alternative explanations for the variability in mean subscale scores across the studies, notwithstanding their alternative Likert scale scoring system which significantly reduced scale scores. These include cultural aspects, type of academic training and differences in the curricular training of university programs. Magalhães et al. also suggested that healthcare providers in European countries would produce lower biomedical but higher psychosocial beliefs because the biopsychosocial model originated in this continent.

If the Magalhães et al., study was scored on a “1-6” Likert scale and increased by a similar amount as those of Victorian chiropractors when re scaled, which appears highly likely as standard deviations are similar between studies, then those scores would fall within the midrange of previous studies. This suggests that practitioner responses are similar across culture and educational training.

Magalhães et al. [14,21] suggested that healthcare professionals whose attitudes and beliefs reflect a stronger biomedical profile are more likely to search for a specific cause of low back pain and prescribe more imaging exams, encourage rest and time off work in an attempt to reduce tissue damage. In contrast to this expectation Victorian chiropractors have been shown to largely adhere to evidence-based guidelines for X-ray referrals [29]. This further suggests that the chiropractic population is not biased toward a biomedical or psychosocial orientation.

The most significantly disparate score is that of the Irish GP’s psychosocial scale, which was 16.3 (SD = 3.1) [18]. The authors removed 9 items which excludes the possibility of comparisons. This is unfortunate as the study of Queally et al. (2008) suggested that training in musculoskeletal medicine in Ireland was inadequate and required urgent attention and data from the PABS.PT may have been useful in identifying educational competency gaps [30].

The biomedical orientation scale internal consistency in this study was found to replicate levels of previous studies. Other studies have reported less favourable internal consistency levels in the psychosocial scale. This study obtained the lowest Cronbach’s alpha to date (0.44). The original study of Ostelo et al. [16] achieved a level of 0.54, which was strengthened by the items added by Houben et al. [17] and achieved a level of 0.68. The items in the psychosocial scale do not appear to capture the psychosocial orientation in Victorian chiropractors. There is a need for the identification of alternative items to more robustly quantify this dimension in the PABST.PT.

While several demographic variables have been found to influence the biomedical orientation in other studies (gender, age, number of years in practice) this study was not able to identify any such relationships. The number of patients treated per week approached significance at the 5% level in relation to the psychosocial scale. This unexpected finding suggests that the practitioner who sees larger numbers of patients per week is more likely to believe that psychosocial factors play a role in patients’ lower back pain. The poor Cronbach’s alpha result for the psychosocial scale may also be a possible explanation for this trend and casts uncertainty over this finding.

This study did not replicate past studies which have identified relationships with the biomedical scale and GPs and physiotherapists in early and late working life stages. This study’s population was biased toward those chiropractors in “mid-life” and a targeted study seeking to recruit larger numbers from those age groups may be required to reveal if this pattern exists in Victorian chiropractors.

The results of this study and previous studies have not found any relationship to the PABS.PT psychosocial subscale and demographic variables. The lower scores for internal consistency may be a possible explanation. Future studies exploring alternative items may overcome this concern and add to the potential utility of the PABS.PT.

### Limitations of this study

This study was limited by its low response rate. Comparison to the national workforce data suggests that this studies sample approximated it, aside from the younger age groups. Nonetheless non-responders may be aligned to those of high biomedical and low psychosocial patterns but only a larger sample would clarify this possibility.

When compared to the national chiropractic data this sample was underrepresented by younger aged practitioners with fewer years in practice. This will be the “longest practicing” portion of the population who this study focused on and will subsequently have the potential to deliver suboptimal care for many years. A base-line measure would allow an insight into the changes in attitudes across their working lifespan and to determine if future generations of manual therapists are improving in their ability to deliver best practice guideline based care.

This poor internal consistency of the psychosocial scale suggests that the survey items are not capturing this dimension in the chiropractic population. The development of alternative items remains an objective for future research.

A strength of this study was the single professional practitioner base of participants. It enabled a comparison to previous studies comprised of various populations. Chiropractic practitioners do not appear to differ significantly from other health care providers. Thus the PABS.PT, in particular the biomedical scale may offer some utility in identifying non guideline based chiropractitioners.

## Conclusions

Practitioner attitudes and beliefs have been shown to be associated with clinical outcomes for patients with low back pain [12]. This study is the first to explore chiropractors’ attitudes and beliefs in isolation to determine whether their attitudes and beliefs are consistent with previous studies of mixed practitioner populations and differing cultural backgrounds. Past studies have suggested that chiropractors may hold extreme views which bias toward a biomedical emphasis. The results of this study suggest that the sample of Victorian chiropractors demonstrated similar levels to that of other health professions from differing cultures and educational backgrounds as measured on the PABS.PT. Future research is needed to identify items to improve the internal consistency of the psychosocial scale. Once achieved a larger study may be conducted to explore if the non-findings of age, gender and years in practice are robust.
